# Comparing Multidrug-Resistant (MDR) and non-MDR Patients in Intensive Care Unit: Outcomes and Challenges

**DOI:** 10.12669/pjms.41.9.12029

**Published:** 2025-09

**Authors:** Kaleem Ullah Toori, Ramsha Ghazal Arshad, Javeria Rahim

**Affiliations:** 1 Kaleem Ullah Toori, MBBS, MRCP(UK), FRCP (Glasgow) KRL Hospital, Islamabad, Pakistan; 2 Ramsha Ghazal Arshad, MBBS KRL Hospital, Islamabad, Pakistan; 3 Javeria Rahim, MBBS KRL Hospital, Islamabad, Pakistan

**Keywords:** Multidrug-resistant pathogens, Intensive care unit, Risk factors

## Abstract

**Background and Objectives:**

Antibiotic resistance is a major global health issue, particularly in intensive care units (ICUs), where critically ill patients are more vulnerable to multidrug-resistant (MDR) infections. The objective was to identify key factors contributing to the emergence of MDR pathogens in ICU patients.

**Methodology:**

This prospective cross-sectional observational study was conducted at the medical ICU of Khan Research Laboratories (KRL) Hospital in Islamabad, Pakistan, from September 2022 to August 2023. A total of 750 adult patients (aged ≥18 years) were included in the study, divided into two groups: 375 patients with MDR infections (Study group) and 375 patients without MDR infections (Control group). Clinical and laboratory data were collected, including patient history, comorbidities, recent hospitalizations, and results from culture and antibiotic susceptibility testing and analysed using multivariate regression analysis.

**Results:**

From a total of 750 patients, 375 were in each group, mean age was 59.5 ±15.9, with 405(53%) males. Patients with comorbidities, low Hb, renal or coagulation impairment were more in the MDR group. Age, previous history of cardiovascular disease, cerebrovascular disease mechanical ventilation, high CRP and number of indwelling lines and coagulation dysfunction were significant risk factors linked to mortality.

**Conclusion:**

Modifiable factors like indwelling lines and use of mechanical ventilation play a critical role in the development of MDR infections in ICUs. Addressing these factors through targeted interventions may help reduce the burden of MDR pathogens and improve patient outcomes in critical care settings.

## INTRODUCTION

Antibiotic resistance is a critical global health challenge of the 21st century, with the World Health Organization (WHO) identifying it as a major threat to health, food security, and development.^1^ Multidrug-Resistance (MDR) is highlighted as a significant socioeconomic burden over the past century and a key area for future healthcare research advancements.^2^,^3^

Intensive Care Units (ICUs) provide essential care for patients with severe illnesses, but their vulnerable populations are at high risk for multidrug-resistant (MDR) infections. These infections lead to increased mortality rates, longer hospital stays, and significant economic strain on healthcare systems.^4^,^5^ MDR complicates treatment options, often forcing the use of less effective, last-resort antibiotics with more side effects. Research across disciplines has focused on the efficacy, safety, and recovery of patients affected by MDR.^3^,^5^,^6^

MDR in ICUs arises from complex factors, including bacterial mutations and gene transfer, exacerbated by antibiotic overuse. The high use of antibiotics creates selective pressure that fosters resistant strains. Environmental factors also play a crucial role, with contaminated surfaces, medical devices, and healthcare workers acting as reservoirs for resistant pathogens.^7^ Rigorous cleaning protocols are essential due to the frequent contamination of high-touch surfaces. Moreover, ventilation systems and water supplies can harbor pathogens. High patient turnover increases the risk of spreading resistant strains, while transfers between wards or hospitals complicate infection control by facilitating the dissemination of these pathogens across healthcare settings.^6^,^7^

The factors contributing to MDR in ICUs are complex, involving both patient characteristics and healthcare practices. These elements create an environment conducive to the growth of resistant microorganisms and facilitate the spread of resistance to other facilities.^8^ This is particularly evident in ventilator-associated pneumonia, where inadequate initial antibiotic therapy can worsen patient outcomes. Infections from resistant organisms can severely impact prognosis if the initial treatment is ineffective, highlighting the need for effective antimicrobial stewardship and rapid diagnostics in ICUs.^9^ Additionally, common ICU medications, such as sedatives and muscle relaxants, can increase infection risks, and inappropriate antibiotic selection is linked to higher morbidity and mortality rates.^10^

Key factors influencing MDR in ICUs, identified in various studies, include advanced age, severity of illness, prolonged ICU stays, prior antibiotic use and the presence of indwelling devices like central venous catheters and mechanical ventilators.^7^,^8^,^9^,^10^ Common antibiotic-resistant pathogens in ICUs include Methicillin-Resistant Staphylococcus Aureus (MRSA), Vancomycin-Resistant Enterococci (VRE), and Carbapenem-Resistant Enterobacteriaceae (CRE).^11^

Recent studies in Pakistan have showed a rising prevalence of MDR infections in hospitalized patients. One study done by Faiza et al showed a prevalence of multi-drug resistant infections in ventilated patients (26.2%) of which 88% were Acinetobacter *sp*.^12^ Similarly a meta-analysis done across Pakistan showed a significant pooled proportion of ESBL acquisition, of which Islamabad ranked the highest amongst other cities.^13^A systematic review showed that ICU-acquired infection and antimicrobial resistance are 10% more prevalent in lower- middle income countries (LMICs) compared to high-income countries (HICs) with a mortality rate of 33.6% in LMICs versus less than 20% in HICs.^14^

This study aimed to identify factors associated with the acquisition of multidrug-resistant (MDR) infections in local ICUs, to compare them with non-MDR infections, and examine their relationship with mortality to inform strategies for managing and preventing future adverse outcomes.

## METHODOLOGY

This prospective cross-sectional observational study was carried out at the medical intensive care unit of KRL Hospital, Islamabad, Pakistan from September 2022 to August 2023. Sample size was calculated as per following assumptions: Precision= 5%, Prevalence of MDRO acquisition 41%, Population size= infinite. With 95% confidence interval, the estimated sample size (n) =372.^15^

### Inclusion & Exclusion Criteria:

A total of 375 patients of ≥18 years of age admitted in the ICU and diagnosed with MDR infections were included in the Study group through probability, consecutive sampling. Another 375 patients of ≥18 years of age admitted in ICU and not reported with MDR infections were included as Control group. The exclusion criteria were patients with ICU stays less than 48 hours, those transferred from other ICUs, pregnant or post-partum patients or those in surgical ICUs.

### Operational definitions:

### Multidrug resistant organisms (MDROs):

were defined as organisms, particularly bacteria, resistant to at least one or more antibiotics in three or more antimicrobial categories.^16^

### APACHE II:

a tool used to assess the severity of illness in critically ill patients particularly in the context of ICU patients and provides a numerical score that correlates with the risk of mortality.

Comorbidities:

a) Hypertension- ABPM> 140/90mmHg or already on > antihypertensives.
b) Diabetes: HbA1c> 47mmol/mol or on oral hypoglycemics/insulin.
c) Ischemic heart disease: history of PCI/ angina/prior MI.
d) Cerebrovascular accidents: prior history of stroke with or without residual weakness/ Transient ischemia attack.
e) DCLD: known cirrhosis secondary to any cause with history of ascites/encephalopathy/ GI bleed.
f) Respiratory: known chronic lung conditions including chronic obstructive lung disease, asthma, interstitial lung disease, bronchiectasis- on treatment.
g) Malignancy: cancer diagnosed within previous six months, recurrent, on treatment.
h) CKD: eGFR<60ml/min for > 3months, proteinuria>30mmol, structural changes on imaging.

All the demographics, medical history and clinical findings of the participants were recorded on a predesigned pro forma. This included admission details (reason for ICU admission and prior hospitalizations), APACHE II score at the time of admission, duration of antibiotic used prior to admission, invasive procedures and devices (such as central venous catheters, urinary catheters, and endotracheal intubation), ICU interventions (MV, and vasopressor use). Comorbid medical conditions were further stratified into groups namely respiratory, hypertension, ischemic heart disease, cerebrovascular accidents, malignancies and decompensated liver disease. Microbiology data encompassed all cultures obtained, organisms isolated, and antibiotic susceptibility results were also recorded. Specimen collection, culture techniques, and antibiotic susceptibility testing was done as per local protocols in accordance with the microbiologist in KRL hospital including disc diffusion and dilution susceptibility tests. Antibiotic resistance was declared when infections occurring after 48 hours of ICU admission caused by antibiotic-resistant pathogens.^16^,^17^

The primary outcome of the study was to determine the factors affecting the emergence of multidrug-resistant pathogens in ICU settings and their association with mortality.All data was analyzed using the SPSS 23 software. Descriptive analysis was used to summarize patient characteristics, medical history and clinical findings. Kolmogorov Smirnov test was applied to assess the normality of the data. Categorical variables were compared using Chi-square test while continuous variables, were analyzed using Mann Whitney U test. Logistic regression analysis was applied to identify independent risk factors for the emergence of MDR pathogens, adjusting for potential confounders. Statistical significance was taken at p < 0.05.

### Ethical Approval:

It was obtained from the Institutional Review Board prior to initiating this research (Ref: KRL-HI-PUB-ERC/Sep22/15-B; Dated: September 15, 2022).

## RESULTS

The total number of patients included in the study were 750, of which 402 (53.6%) were females, 431(57.5%) were hypertensive, 387 (51.6%) diabetics, 221(29.5%) had IHD, 132 (17.6%) had chronic kidney disease, 264 (48.5%) had a pre-existing lung condition, 32(4.3%) had DCLD, 86(11.5%) had a previous history of CVA and only 22 (2.9%) had history of malignancy. Table-I shows rest of the baseline characteristics of the overall population and further stratification amongst the two study groups. Mean age of the sample was 59.5 ± 15.7 and mean duration of hospital stay was 10.1 ± 8.6 days with an overall mortality of 172 (22.9%) of which 139 (81%) belonged to the MDR group. The two groups (MDR vs non-MDR) showed a slight male predominance in MDR group but this difference was not statistically significant. Similarly, elderly patients, hypertensive, diabetics, those with chronic lung conditions, previous use of antibiotics, indwelling lines, anemia, thrombocytopenia, high CRP, deranged renal functions were more prevalent in MDR group and duration of hospital stay was also more in the same cohort. Out of the overall 134 patients who underwent mechanical ventilation, 111 (82.8%) were in the MDR group and this difference was statistically significant.

**Table-I:** Baseline characteristics of the study population.

Variable	Overall group	Mdr	Non-mdr	Pearson’s chi square	P=value
Age	59.5 ±15.9	62.1 ± 16.9	57.8 ±13.8		<0.01
***Gender***					
Male(%)	402(53.6)	190(50.6)	212(56.5)	2.59	0.12
Female(%)	348(46.4)	185(49.3)	163(43.5)		
***Comorbidities***					
Hypertension(%)	431(57.5)	259(60)	172(40)	41.3	<0.01
Diabetes(%)	387(51.6)	253(65.3)	134(34.7)	75.6	<0.01
IHD(%)	221(29.4)	131(59.2)	90(40.8)	10.8	0.001
CKD(%)	132(17.6)	108(81.8)	24(18.2)	64.8	<0.01
DCLD(%)	32(4)	16(50)	16(50)	0.000	1.00
CVA(%)	86(11.4)	49(56.9)	37(43.1)	1.89	0.27
Malignancy(%)	22(2.9)	7(31.8)	15(68.2)	2.99	0.13
Respiratory(%)	364(48.5)	215(59)	149(41)	23.2	<0.01
Previous hospital admission in last 6 months(%)	174(23.2)	131(75.2)	43(24.8)	57.9	<0.01
Number of antibiotic used in last 6 months	1.91 ±0.9	2.3 ±0.99	1.52 ±0.56		<0.01
Days of antibiotic use	8.5 ± 3.4	10.3 ±3.7	6.6 ±1.6		<0.01
Mechanical ventilation(%)	134(17.8)	111(82.8)	23(17.2)	70.4	<0.01
Number of indwelling lines	1.87 ±1.7	2.35 ±1.6	1.4 ±0.76		<0.01
Duration of indwelling lines	9.79±10	15 ±11.9	4.5 ±2.1		<0.01
APACHE II score	25.6±2.3	20.98±2.7	20.56±2.6		0.03
Inotropes(%)	192(25.6)	155(80.7)	33(19.3)	111.1	<0.01
Steroids(%)	129(17.2)	77(59.6)	52(40.4)	5.85	0.02
Hemoglobin	11.1 ±1.9	16 ±10.7	11.5 ±1.6		<0.01
WCC>10,000	14.8 ±4.99	15.1 ±5.7	14.6 ±4.1		0.27
Platelets	243 ±78	235 ±0.8	251 ±72.6		<0.01
CRP	76 ±7	142 ±85.6	106 ±59		<0.01
Renal dysfunction(%)	266(35.4)	182(68.4)	84(31.6)	55.9	<0.01
Coagulation dysfunction(%)	27(3.6)	16(59)	11(41)	0.96	0.43
Duration of hospital stay	10.1±8.6	14.7 ±10.1	5.5 ±2.1		<0.01
Mortality(%)	172(22.9)	139(80.9)	33(19.1)	84.7	<0.01

*Ischemic heart disease, ** Decompensated Chronic Liver Disease, *** Cerebrovascular accidents, **** Chronic kidney disease.

Univariate analysis was done and variables with a *p-*value of <0.05 were selected for multivariate logistic regression analysis. Table-II. The final model revealed that the age, previous cardiovascular disease, previous history of CVA, number of indwelling lines, mechanical ventilation, high CRP, coagulation dysfunction was independently associated with increased mortality in the ICU in the MDR cohort. Table-III.

**Table-II:** Univariate analysis.

Variables	Or (95%ci)	P-value
Age	1.04(1.024,1.054)	<0.01
Gender	0.84(0.56,1.29)	0.44
Hypertension	2.21(1.36,3.60)	0.001
Diabetes	1.18(0.75,1.86)	0.46
IHD	2.63(1.63,4.08)	<0.01
CKD	2.26(1.43,3.57)	<0.01
Respiratory	2.15(1.89,2.23)	0.03
CVA	3.85(2.04,7.25)	<0.01
DCLD	1.02(0.36,2.86)	0.97
Malignancy	2.65(0.92,7.62)	0.07
Previous antibiotic use	1.96(1.55,2.47)	<0.01
Duration of antibiotic use	1.06(0.99,1.12)	0.06
Previous hospital admission in 6 months	2.05(1.32,3.17)	0.001
Mechanical ventilation	7.37(4.53,12.1)	<0.01
Number of Indwelling lines	4.04(3.03,5.38)	<0.01
Duration of indwelling lines	1.01(0.99,1.03)	0.17
Inotropes	1.15(0.75,1.76)	0.51
Steroids	0.96(0.57,1.62)	0.88
Hemoglobin	0.79(0.71,0.89)	<0.01
TLC>10,000	1.009(0.97,1.047)	0.62
Platelets	1.00(0.99,1.002)	0.78
CRP	1.005(1.003,1.008)	<0.01
Renal dysfunction	2.61(1.69,4.02)	<0.01
Coagulation dysfunction	8.01(2.24,28.6)	0.001
Duration of hospital stay	1.01(0.99,1.03)	0.18

**Table-III:** Multivariate logistic regression analysis.

Variable	Regression coefficient	or (95% ci)	p- value
Age	0.49	1.05(1.02,1.07)	<0.01
Hypertension	-0.43	0.96(0.46,1.98)	0.91
IHD	0.87	2.38(1.27,4.46)	0.007
CKD	-0.37	0.69(0.29,1.60)	0.38
CVA	0.99	2.7(1.11,6.58)	0.03
Respiratory	0.35	0.77(0.39,1.49)	0.44
APACHE II score	0.61	1.01(0.97,1.04)	0.77
Hospitalization in last 6 months	0.68	1.97(0.95,4.12)	0.69
Mechanical ventilation	2.01	7.48(2.54,22.4)	<0.01
Number of prior antibiotic use	0.21	1.23(0.85,1.78)	0.27
Number of indwelling lines	0.99	2.69(1.69,4.29)	<0.01
Hemoglobin	-0.11	0.89(0.74,1.08)	0.26
CRP	0.006	1.006(1.002,1.01)	0.002
Renal dysfunction	-0.51	0.59(0.25,1.42)	0.24
Coagulation dysfunction	2.49	12.1(2.27,64.2)	0.003

## DISCUSSION

Antibiotic resistance has emerged as a critical global health crisis, with the rise of multi-drug resistant (MDR) infections contributing to increased morbidity and mortality. Factors driving this crisis include the overuse of antibiotics, multiple comorbid conditions, frequent hospitalizations, and invasive procedures.^17^ In this study, we examined the independent risk factors for acquiring MDR infections and their association with mortality in ICU patients, comparing both MDR and non-MDR cases. Our analysis revealed that age, previous history of cardiovascular disease, mechanical ventilation, high CRP and number of indwelling lines and coagulation dysfunction were significant risk factors linked to mortality. We observed that the mean age of the population was higher as compared to that of the non-MDR group (62.1 vs 57.8, *p<0.01*).

In this study, there was no difference in MDR acquisition between males and females. This is in contrast to previous researches that showed that men had a higher incidence of MDR infections.^18^ This difference may be attributed to the equal distribution of genders between the two groups and a nearly equal likelihood of antibiotic exposure within the community.

Our study also found a higher prevalence of pre-existing comorbid conditions among patients in the MDR group, including hypertension, diabetes, ischemic heart disease (IHD), chronic kidney disease (CKD), and chronic respiratory disorders. This may be explained by the aging population, who, over time, tend to accumulate multiple comorbidities and require more frequent healthcare interventions. Similarly, these patients are often immunocompromised, leading to increased antibiotic use and more frequent presentations to acute care settings.^19^,^20^ A post hoc analysis by Zorrilla et al showed that about 93% of the study population (MDR) had an underlying comorbid condition and this difference was statistically significant.^21^ Hypertension, diabetes and chronic lung conditions were amongst the most frequent comorbid illnesses in out cohort and malignancies being the least common. This difference may be attributed to the lack of local oncology services and the early transfer of these patients to specialized facilities.

Our study also demonstrated that patients with a history of hospitalization and antibiotic use within the past six months had a higher risk of developing multidrug-resistant (MDR) infections.^22^,^23^ Further analysis of the study revealed that intensive care unit (ICU) interventions also predisposed patients to multidrug-resistant (MDR) infections, consistent with findings from previous research.^19^,^24^-^26^ Biochemical parameters showed that low hemoglobin, thrombocytopenia, high CRP, renal and coagulation dysfunction was more in MDR group. Anemia in the ICU is often multifactorial and has been associated with increased mortality, either due to impaired oxygen delivery or as a consequence of chronic inflammation.^27^ Similarly, renal dysfunction in ICU patients can result from multiple factors, including direct injury from toxic metabolites and drug-related effects. Kidney injury may further contribute to a chronic inflammatory state leading to increased mortality, as supported by existing literature.^28^,^29^

Our study did not show any significant difference in APACHE II score amongst the two groups which contradicts its use for determining adverse outcomes. This may be attributed to improved survival rates over recent years among patients with conditions requiring ICU interventions. As a result, these scoring systems might overestimate or underestimate the actual probability of mortality. Newer scores are now being employed to assess and quantify the risk of adverse outcomes in ICU patients.^30^

Coagulation abnormalities and inflammation are well-recognized to be interconnected, frequently manifesting together in critically ill patients. This relationship is multifactorial, involving the activation of enzymes that initiate the coagulation cascade, and is associated with the development of disseminated intravascular coagulation (DIC) and increased mortality in affected patients.^31^ This is consistent with a 2019 study conducted in Italy, which demonstrated that coagulation dysfunction was independently associated with increased mortality in patients with multidrug-resistant (MDR) infections.^32^ Multivariate analysis showed that advancing age, cardiovascular disease, cerebrovascular disease, mechanical ventilation, number of indwelling lines, high CRP and coagulation dysfunction were independent risk factors linked to mortality in MDR patients in ICU.

### Strength:

The strength of our study lies in using simple, readily available patient characteristics and biochemical markers to predict adverse outcomes in multi-drug resistant infections in an ICU setting.

### Limitations:

Conducting this study in a single ICU setting may limit the generalizability of the findings and potentially underemphasize the broader association between risk factors for MDR acquisition and their impact on patient outcomes and may introduce bias. Follow up parameters and response to treatment were not assessed. However, we assumed that our sample was representative of the local population. Performing a multicenter ICU study with a larger sample size could help validate these findings and enhance their applicability to the general population. Furthermore, analyzing these variables separately may help clarify the discrepancies noted in the study.

## CONCLUSION

This study identified key risk factors for MDR infections in ICU settings, including patient age, comorbidities, invasive procedures, and coagulation dysfunction. Importantly, previous antibiotic use, MV and indwelling lines emerge as key modifiable factors. By focusing on these factors, healthcare providers can develop targeted strategies to minimize this risk. This approach can offer a promising path to enhance patient outcomes and address the growing challenge of MDR in our ICUs. Further studies including multiple ICUs could help to further explore the relationship between these risk factors and MDR acquisition.

### Authors Contribution:

**KUT:** Conception and design, critical revision of the data, final approval before publication, responsible for the integrity and accuracy of the study.

**RGA:** Acquisition of data, data analysis and manuscript writing.

**JR:** Literature search, Acquisition of data and manuscript writing.
